# Editorial: Reviews in opioid use disorders

**DOI:** 10.3389/fpubh.2024.1467826

**Published:** 2024-08-20

**Authors:** Wendy Walwyn, Kirk E. Evoy

**Affiliations:** ^1^Department of Psychiatry and Biobehavioral Sciences, University of California, Los Angeles, Los Angeles, CA, United States; ^2^Department of Pharmacotherapy and Translational Science, The University of Texas at Austin College of Pharmacy, Austin, TX, United States; ^3^Department of Pharmacy, University Health, San Antonio, TX, United States

**Keywords:** naloxone, kratom, buprenorphine, medications for opioid use disorder, methadone

The exponential increase in opioid use disorder (OUD) to 60 million who battle this disorder worldwide and more than 100,000 who suffer fatal overdose annually is more than an astonishing and sad statistic; it is one for which we need solutions (1). What started as a problem of prescription opioids in the early 2000s and subsequently shifted to heroin, is now melding into combinations of ultra-potent synthetic opioids and other drugs with lethal consequences that are harder to predict and prevent. The only silver lining to this very dark cloud is that this epidemic has fostered an exponential increase in research and innovation around all aspects of this disorder from the cellular mechanisms to new public health approaches and diverse avenues of treatment.

New approaches to prevention and treatment are clearly needed if we are to succeed in reversing this epidemic. We need to consider early stages of the disorder, including the pre-addiction stage, when changes in the dopaminergic reward circuitry could be addressed before OUD becomes an engrained and difficult to treat condition (see Lee et al.). To do so, we would need to be able to recognize and diagnose pre-addiction (2) much as pre-diabetes is a recognizable and treatable disease that can prevent the development of diabetes. There is also a change needed in how we think of those with OUD. This disorder does not just afflict patients in pain with a history of chronic prescription opioid use; in fact, there is limited connection with past opioid prescriptions (2).

We must also rethink our treatment approach. Increasing the availability and use of naloxone, a mu opioid receptor antagonist, available in different formulations to rapidly reverse the effects of opioids is one important step (3), as reviewed by Dahan et al.. However, as the substances involved in overdose change, further discussion about the dose, formulations, and alternatives are necessary. To reverse opioid induced respiratory depression, the dose and delivery of naloxone must be sufficient to displace and reduce mu opioid receptor occupancy by 50%. With increasingly potent opioids being introduced into the illicit drug supply, repeated naloxone administration or higher-dose naloxone formulations may be needed, though this must be balanced with the potential for increased adverse effects or precipitation of uncomfortable opioid withdrawal. Expanding the treatment armamentarium to include additional opioid antagonists, such as the recently approved nalmefene, may further improve our ability to reduce opioid overdose mortality.

While naloxone offers an effective overdose reversal agent, it does not treat the underlying disorder. Medications for opioid use disorder (MOUD) such as methadone, buprenorphine, and naltrexone, are currently the gold standard for OUD treatment (4). Methadone maintenance therapy has been used for many years to treat OUD but there is still work to be done in increasing access and reducing restrictions around its use. Despite recent removal of waiver requirements to prescribe buprenorphine, much work remains to expand access to this lifesaving drug as well (5), especially for vulnerable populations such as those recently released from jail, as reviewed by Chladek and Chui. OUD incidence in this population is far higher than the national average, and for those with OUD, the likelihood of recidivism back into the system is high. MOUD needs to be part of a multi-dimensional treatment recovery program consisting of mobile healthcare, telehealth, peer support specialists, community pharmacists and other support avenues if we are to lessen recidivism.

Though MOUD is the gold standard treatment option for OUD, many people with OUD lack access to treatment, and if they do have access, half discontinue treatment. This is accompanied by a 6-fold increase in mortality in the first 4 months due to enhanced sensitivity to opioid agonists and difficulty by users in assessing drug efficacy (6). A recent multi-million dollar study across 67 intervention sites showed no effect on the number of overdose fatalities between the intervention group receiving different forms of MOUD and the control group (7). Equally another innovative approach, the use of vaccines as immunotherapy for different misused substances has shown less promise than hoped for in clinical trials (8). These findings outline a need for new treatment modalities with alternate mechanisms of action and less risks that will do more than manage the disorder. Treatments that utilize different approaches, such as deep brain stimulation or transcranial magnetic stimulation, and receptors other than the opioid receptors show promise (see Lee et al.). In recent years, there has been renewed interest in a number of older drugs as potential tools to help manage mental health and substance use disorders, such as ketamine or kratom. In this Research Topic, Green et al. highlight kratom. Due to its mild stimulant effects and opioid receptor activity, its use and availability in the USA is increasing (9). Kratom is currently considered a new dietary ingredient by the FDA, so is without guidance as to its medical use or health risks, and research is currently insufficient to recommend its clinical use. However, Green et al. review early pre-clinical data that indicate possible therapeutic potential for kratom-derived products for OUD, and possibly other substance use disorders, though much work is needed to better clarify its potential risks and benefits, identify optimal doses of whole-leaf kratom or extracts of its various active alkaloids, and ensure products are appropriately regulated and labeled.

Despite extensive efforts to address the opioid epidemic, OUD and opioid overdose mortality continue to rise. A better understanding of OUD and societal and public health factors that influence related morbidity and mortality are crucial, as are new treatment modalities to expand the OUD treatment armamentarium. This Research Topic was developed to collate review articles describing current research spanning this broad spectrum of OUD prevention and treatment.

## References

[1] The Lancet Regional Health-Americas. Opioid crisis: addiction, overprescription, and insufficient primary prevention. Lancet Reg Health Am. (2023) 23:100557. 10.1016/j.lana.2023.100557 37497399 PMC10366477

[2] McLellan AT Koob GF Volkow ND. Preaddiction-a missing concept for treating substance use disorders. JAMA Psychiatry. (2022) 79:749–51. 10.1001/jamapsychiatry.2022.1652 35793096

[3] Lai RK Friedson KE Reveles KR Bhakta K Gonzales G Hill LG. Naloxone accessibility without an outside prescription from U.S. community pharmacies: a systematic review. J Am Pharm Assoc. (2022) 62:1725–40. 10.1016/j.japh.2022.07.008 35989151

[4] Oesterle TS Thusius NJ Rummans TA Gold MS. Medication-assisted treatment for opioid-use disorder. Mayo Clin Proc. (2019) 94:2072–86. 10.1016/j.mayocp.2019.03.029 31543255

[5] Hill LG Loera LJ Torrez SB Puzantian T Evoy KE Ventricelli DJ. Availability of buprenorphine/naloxone films and naloxone nasal spray in community pharmacies in 11 U.S. states. Drug Alcohol Depend. (2022) 237:109518. 10.1016/j.drugalcdep.2022.109518 35691255

[6] Santo T Clark B Hickman M Grebely J Campbell G Sordo L. Association of opioid agonist treatment with all-cause mortality and specific causes of death among people with opioid dependence: a systematic review and meta-analysis. JAMA Psychiatry. (2021) 78:979–93. 10.1001/jamapsychiatry.2021.0976 34076676 PMC8173472

[7] Consortium HECS Samet JH El-Bassel N Winhusen TJ Jackson RD Oga EA. Community-based cluster-randomized trial to reduce opioid overdose deaths. N Engl J Med. (2024). 10.1056/NEJMoa2401177 38884347 PMC11761538

[8] Kosten TR. Vaccines as immunotherapies for substance use disorders. Am J Psychiatry. (2024) 181:362–71. 10.1176/appi.ajp.20230828 38706331

[9] Covvey JR Vogel SM Peckham AM Evoy KE. Prevalence characteristics of self-reported kratom use in a representative US general population sample. J Addict Dis. (2020) 38:506–13. 10.1080/10550887.2020.1788914 32657217

